# Irisin: Emerging Therapeutic Targets for Cognitive Impairment-Related Diseases

**DOI:** 10.1017/erm.2025.10014

**Published:** 2025-07-15

**Authors:** Mei Ma, Guangchan Jing, Yue Tian, Ruiying Yin, Mengren Zhang

**Affiliations:** Department of Traditional Chinese Medicine, Peking Union Medical College Hospital, Peking Union Medical College and Chinese Academy of Medical Sciences, Beijing, China

**Keywords:** AD, cognitive impairment, DACD, FNDC5, irisin

## Abstract

**Introduction:**

Irisin is a glycosylated polypeptide hormone derived from muscles that plays a crucial role in learning and memory by promoting the growth of hippocampal neurons, thereby influencing cognitive function.

**Objective:**

Despite increasing evidence, a comprehensive understanding of the exact role of irisin remains elusive, necessitating further research to unravel the complex mechanisms through which irisin influences cognitive function and to explore therapeutic approaches targeting irisin.

**Method:**

A literature review was performed by searching PubMed for articles published between 2012 and 2024, using the keywords ‘fibronectin type III domain-containing 5 (FNDC5)’, ‘irisin’, ‘cognitive impairment’, ‘Alzheimer’s disease’, ‘Age-related cognitive dysfunction’ and ‘Diabetes-associated cognitive dysfunction’, combined with Boolean operators (AND/OR).

**Results:**

This review highlighted the potential impact of irisin on cognitive function in the context of ageing, diabetes and Alzheimer’s disease. The anti-cognitive impairment effects of irisin are associated with the regulation of energy metabolism, insulin resistance, inflammation, oxidative stress, amyloid-beta deposition, synaptogenesis and plasticity. The signalling pathways through which irisin improves cognitive impairment are complex and highly regulated processes, involving multiple signalling pathways such as the adenosine monophosphate-activated protein kinase (AMPK) signalling pathway, mitogen-activated protein kinase (MAPK) signalling pathway, nuclear factor-κB (NF-κB) signalling pathway, ERK-STAT3 signalling pathway, cAMP/PKA/CREB signalling pathway and Nrf2/HO-1 signalling pathway.

**Conclusion:**

This review delves into the positive effects of irisin on cognitive impairment, examines the signalling pathways related to fibronectin type III domain-containing 5 (FNDC5)/irisin and provides future perspectives for research on the anti-cognitive impairment effects of irisin.

## Graphical abstract

**Figure:**
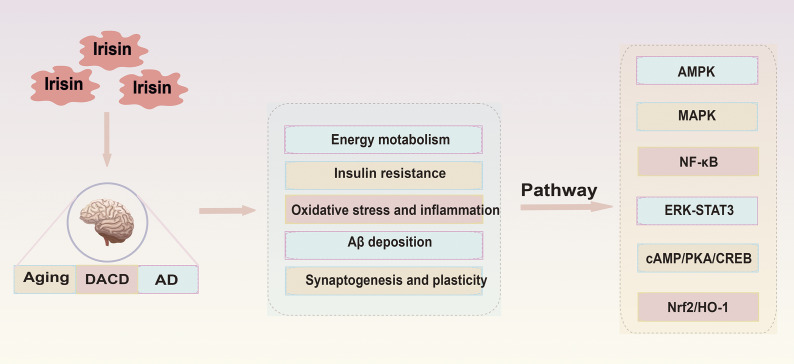

## Introduction

The global incidence of cognitive disorders has sharply increased over the past few decades, particularly among elderly individuals, with mild cognitive impairment affecting 10% to 20% of adults aged 65 and older (Ref. 1). The risk of cognitive disorders increases with age, with males appearing to be at higher risk compared to females (Ref. 1). Alzheimer’s disease (AD) and diabetes have emerged as global epidemics associated with cognitive disorders, imposing substantial social and economic burdens on public health systems worldwide. Consequently, understanding how to maintain and promote cognitive function in the brain and how to delay or prevent cognitive decline has become a significant challenge. Since its discovery, irisin has attracted considerable attention due to its biological functions, including promoting the browning of white adipose tissue, accelerating energy consumption, regulating energy metabolism and improving insulin resistance (Ref. 2). A large body of research indicates that irisin has a therapeutic potential for various chronic diseases, including obesity, diabetes, bone metabolism and cardiovascular diseases (Ref. 3). Furthermore, irisin can cross the blood–brain barrier (BBB) and is widely distributed in regions of the brain, such as the cortex, hippocampus, striatum and hypothalamus, where it plays crucial roles in normal physiological processes (Ref. 4). Increasingly, researchers are considering irisin an important therapeutic target for neurodegenerative diseases, given its close association with cognitive impairments. A comprehensive understanding of the relationship between irisin and cognitive functions in the brain could facilitate the development and clinical application of irisin-based therapies.

## Method

A literature review was performed by searching PubMed for articles published between 2012 and 2024, using the keywords ‘FNDC5’, ‘irisin’, ‘cognitive impairment’, ‘Alzheimer’s disease’, ‘Age-related cognitive dysfunction’ and ‘Diabetes-associated cognitive dysfunction’, combined with Boolean operators (AND/OR). Filters were applied to include only English-language articles, animal models, human studies and peer-reviewed original research. Initial results were screened by title/abstract for relevance to FNDC5/irisin and cognitive impairment, followed by full-text review of articles. Duplicates and studies lacking mechanistic data were removed.

## Background on Irisin

Irisin is a glycosylated polypeptide hormone of muscular origin, first discovered by Boström and colleagues in 2012 (Ref. 2). It is produced through proteolytic cleavage of the fibronectin type III domain-containing 5 (FNDC5) protein and released into the bloodstream (Ref. 2). The gene encoding irisin is located on human chromosome 1p35.1 (Ref. 5). Irisin is a protein consisting of 112 amino acids, which are 100% identical across rats, mice and humans, indicating highly conserved functionality potentially mediated by cell surface receptors (Ref. 2). Although irisin is primarily sourced from muscle and adipose tissue, its biological functions are exerted through specific receptors known as integrins, which are widely distributed throughout the body, including in adipose tissue, skeletal muscle, liver and the central nervous system (Refs. 6, 7). Currently, the receptors of irisin have not been fully determined. Research suggests that irisin binds to proteins of the αV integrin class, with αV/β5 integrin demonstrating the highest binding affinity (Refs. 8, 9). The extracellular chaperone heat shock protein-90 (Hsp90) acts as an activator that ‘opens’ the αV/β5 integrin receptor, facilitating high-affinity binding of irisin and effective signal transduction (Ref. 10). Interestingly, FNDC5 and irisin can be detected in both peripheral circulation and the central nervous system (Ref. 11). However, it remains unclear whether irisin in cerebrospinal fluid originates from the central nervous system or peripheral circulation. While some studies indirectly suggest that circulating irisin can cross the BBB, specific attributes of irisin’s BBB permeability and direct evaluation of membrane transport mechanisms require further investigation. After a decade of in-depth research on irisin, many questions remain unanswered. First, there are still shortcomings in the detection and quantification methods of irisin, with debates focused partly on the specificity of using anti-FNDC5/irisin antibodies for protein identification. Irisin’s apparent molecular weight ranges from 10 to 32 kDa, similar to that of FNDC5, making it difficult to distinguish between FNDC5 and irisin in immunoblots, where both may coexist (Refs. 12, 13). Currently, there are no confirmed reference values for irisin available for rodents or humans. Second, controversies persist regarding the expression patterns of FNDC5 precursor protein and irisin in humans and rodents, specifically concerning the annotated ATG start codon, which mutates to ATA in humans, preventing translation of the full-length protein as seen in most mammals, including mice. Recent studies have found that FNDC5 is translated from an upstream cATG and cleaved to produce a 34-kDa glycosylated protein (Ref. 14). However, the role of the mutated start codon in human FNDC5 and the mechanism of extracellular domain cleavage remain to be explored.

## Evidence of irisin’s impact on cognitive impairment

**Figure 1.**
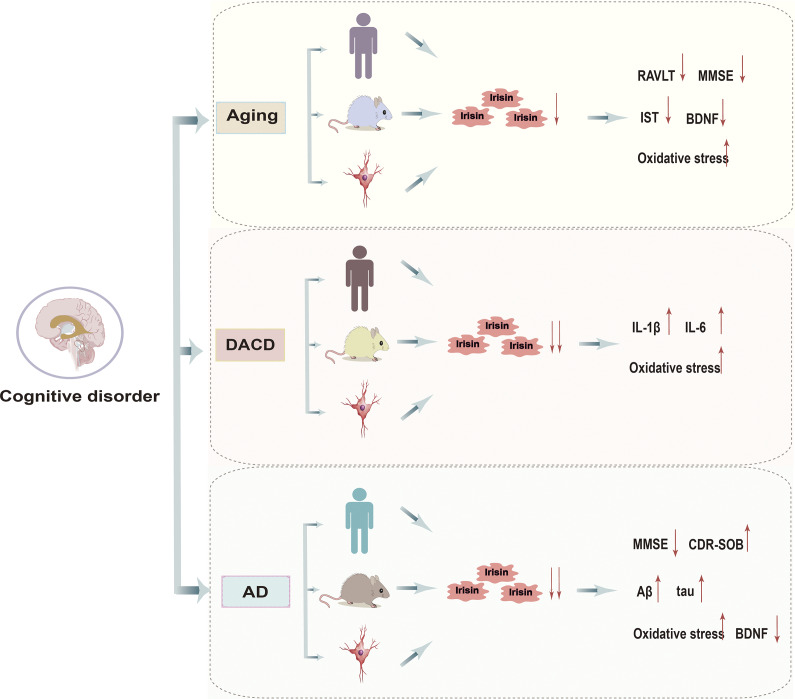
Evidence of Irisin’s impact on cognitive impairment.

Irisin is essential for learning and memory. This section reviews its potential effects on cognitive function in the context of ageing, diabetes and AD (Figure 1).

### Age-related cognitive dysfunction

Aging is an inevitable natural process. With improvements in living conditions, the issue of ageing societies has become increasingly severe. It is estimated that by 2050, the global population aged 60 and over will double to reach 2.1 billion (Refs. 15, 16). Human ageing causes changes in brain function, leading to cognitive impairment. Age-related cognitive dysfunction significantly impacts the lives of elderly individuals and imposes heavy burdens on their families and society (Ref. 15). The mechanisms underlying age-related cognitive dysfunction are not yet fully understood. Studies have found that levels of irisin in cerebrospinal fluid correlate with age, and higher irisin levels have beneficial effects on several cognitive processes, including language recognition, spatial and episodic memory (Refs. 17–20). Clinical studies indicate a significant positive correlation between irisin levels and scores on the Rey Auditory Verbal Learning Test (RALVT), Mini-Mental State Examination (MMSE) and Isaac’s Set Test (IST), which are used to assess long-term language memory and learning abilities (Refs. 19, 20). Moreover, irisin levels show a significant negative correlation with executive function (Ref. 17). Furthermore, research suggests that age-induced cognitive impairment is primarily associated with reduced expression of FNDC5/irisin in the hippocampus (Ref. 21), and recombinant irisin can significantly improve age-related cognitive dysfunction. Similarly, another study confirms that genetic deletion of FNDC5/irisin impairs age-related cognitive dysfunction, partly due to alterations in neurogenesis in the hippocampus (Ref. 4). Recent studies have found that upregulation of FNDC5/irisin in specific brain regions, particularly CA1, may positively affect age-related cognitive functions, especially long-term memory (Ref. 22). This effect may occur through binding to integrin αV/β5 receptors, promoting the secretion of BDNF in hippocampal neurons, thereby enhancing synaptic plasticity. Additionally, recent research shows that irisin administration can counteract astrocyte ageing and improve cognitive decline in P301S mice (Ref. 23). Mechanistically, irisin stimulates the expression of mitochondrial transcription factor A (TFAM), a major regulator of mitochondrial respiratory chain biogenesis, effectively inhibiting astrocyte ageing and enhancing oxidative phosphorylation (OXPHOS) (Ref. 23). Despite these findings, the exact role of irisin in age-related cognitive dysfunction remains incompletely understood. Future studies are needed to comprehensively elucidate the mechanisms by which irisin influences the evolution and progression of age-related cognitive dysfunction.

### Diabetes-associated cognitive dysfunction (DACD)

DACD is a complex complication of diabetes affecting the central nervous system, which is mainly manifested as memory loss, easy distraction, difficulty in focusing on a task for a long time (such as reading and talking) and selective attention deficit (such as difficulty in screening important information from interfering information) (Refs. 24, 25). In recent years, DACD has become a significant area of research interest, with numerous epidemiological studies identifying diabetes as a risk factor for cognitive decline. Its pathological features encompass various aspects, including insulin resistance, deposition of amyloid β-protein (Aβ), neuroinflammation and more (Refs. 25–27). Clinical studies have found that individuals with type 2 diabetes mellitus (T2DM) exhibit significantly lower serum levels of irisin compared to patients without diabetes (Refs. 28, 29). Overexpression of irisin significantly increases cellular vitality under high glucose stress and reduces apoptosis (Ref. 30). Additionally, as chronic complications of T2DM progress, circulating irisin levels gradually decrease, showing a negative correlation between irisin levels and the severity of chronic complications (Refs. 31, 32). Therefore, some researchers propose that irisin could serve as a biomarker for DACD. The improvement of DACD by irisin may be associated with both astrocyte activity and the cascade reaction of neuroinflammation. Studies have shown that irisin prevents memory and cognitive deficits induced by streptozotocin in mice through modulation of STAT3 and inflammatory damage (Ref. 33). Furthermore, irisin may reduce hippocampal tissue and cerebrospinal fluid levels of interleukin-1 beta (IL-1β) and interleukin-6 (IL-6) and upregulate the expression of protective factor MUC3 to counteract damage and inflammation by modulating JAK/STAT in diabetes mellitus (DM) mice astrocyte activation (Refs. 30, 31). On the contrary, other studies have not observed a relationship between irisin and IL-6 or tumour necrosis factor-α (TNF-α) (Ref. 34). Additionally, research has found that a significant up regulation in circulating irisin was found in obese individuals, which was even higher in individuals with impaired fasting glucose or diabetes (Ref. 35). These discrepancies may arise from multiple methodological and biological factors: first, sampling timepoints missing biphasic regulatory windows (acute IL-6 elevation versus chronic TNF-α suppression); Second, hyperglycaemia may induce glycosylation of integrin αV/β5 receptor, which reduces the binding force of irisin. Even if circulating irisin increases, it cannot effectively regulate inflammatory factors. Besides, the interference of platelet-derived IL-6 in serum samples will also affect the accuracy of the results (Refs. 34, 35). To improve consistency, we recommend Ethylenediaminetetraacetic acid (EDTA)-plasma, multi-timepoint designs and receptor function assessment. While we strived to include all relevant studies, the interpretation should be tempered by potential publication bias and methodological heterogeneity across experiments. Currently, the biological mechanisms of irisin in DACD remain unclear. Thus, more systematic studies are needed to clarify the role of irisin in DACD.

### Alzheimer’s disease (AD)-related cognitive dysfunction

AD is the most common form of dementia, characterized by episodic short-term memory impairment in the early stages, with relatively preserved long-term memory. As AD progresses, executive functions such as judgement, problem-solving and organisational abilities become increasingly impaired, accompanied by deficits in visuospatial skills and language functions. The pathogenesis of AD is complex and may involve interactions such as Aβ deposition, neurofibrillary tangle formation, neuroinflammation and oxidative stress (Ref. 36). Irisin has shown potential in improving cognitive function, particularly in relation to AD-related cognitive dysfunction. Clinical research indicates a significant positive correlation between serum irisin levels and overall cognition, as well as episodic memory performance in adults who are at risk of AD (Ref. 37). Reduced serum irisin levels may contribute to neurocognitive deficits observed in obese individuals with a genetic risk for AD during visuospatial working memory tasks. Cerebrospinal fluid irisin levels correlate positively with MMSE scores and negatively with Aβ42 levels, although no correlation was found between irisin and total tau, a marker of neurodegeneration (Ref. 38). Basic research has revealed reduced expression of FNDC5 not only in the hippocampus but also in the frontal cortex of patients with AD (Ref. 4). Genetic deletion of FNDC5/irisin impairs cognitive function in AD, while peripheral administration of irisin effectively mitigates cognitive decline in AD mouse models (Ref. 4). Furthermore, hippocampal FNDC5/irisin expression may negatively correlate with AD-related neuropathology, with irisin signalling involved in transient extracellular signal-regulated kinase (ERK) phosphorylation, increased extracellular brain-derived neurotrophic factor (BDNF) and prevention of AβO-induced oxidative stress (Ref. 39). Importantly, higher plasma irisin levels are associated with late-stage atrophy of the hippocampus, frontal and temporal cortices in AD participants, suggesting the concept of ‘irisin resistance’, where peripheral circulating irisin may fail to exert its normal effects after the onset of AD (Ref. 35). Interestingly, irisin’s therapeutic effects appear to be specific to female htau mice. Irisin treatment significantly reduces ptau and TNF-α levels in the hippocampus and serum of female transgenic htau mice but does not alter ptau levels in the hippocampus of male transgenic htau mice and seems to enhance both neural and systemic TNF-α levels (Ref. 40).

## The potential mechanism of irisin in improving cognitive impairment

**Figure 2.**
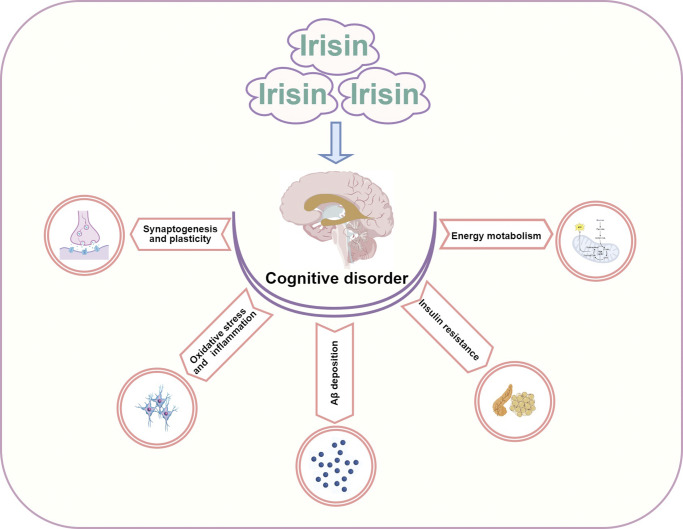
The potential mechanism of Irisin in improving cognitive impairment.

Irisin has a protective effect in restoring memory and cognitive function impairments, opening new avenues for research into cognitive disorders. However, the understanding of how irisin promotes brain physiology and affects disease risk remains limited. Here, we analyse the potential mechanisms of irisin in improving cognitive impairment, focusing on energy metabolism, insulin resistance, Aβ deposition, oxidative stress and inflammation, synaptogenesis and plasticity (Figure 2 and Table 1).

**Table 1.** The potential mechanism of irisin in improving cognitive impairment

Potential mechanism	Disease	Model	Phenomenon	Reference
Energy metabolism	DM	C57BL/6 Mice[HFD + STZ]	FBG↓, HbA1c↓, serum insulin↑	(Ref. 44)
Insulin resistance	DM	C57BL/6 Mice[HFD + STZ] + MIN6 cells	Glu↓, GSIS↑, IRS↓, PI3K↑, AKT↑	(Ref. 48)
Oxidative stress and inflammation	–	NSC–34 cells	SOD2↑, TRX2↑	(Ref. 52) (Ref. 53)
	DM	C57BL/6 Mice[HFD + STZ] + MIN6 cells	NF-κB↓, TNF-α↓, IL–1β↓, IL–6↓,	(Ref. 56)
	DM	C57BL/6 Mice[STZ]	Cognitive Dysfunction↓, STAT3↓, IL–1β↓, IL–6↓,	(Ref. 52)
Aβ deposition	AD	3D cell culture model	NEP↑, Aβ↓ IL–2↓, IL–6↓	(Ref. 33)
	AD	HEK293 cells (APP)	BACE1↓,Aβ↓	(Ref. 64)
Synaptogenesis and plasticity	–	Primary cortical and hippocampal neurons	Cognitive Dysfunction↓, BDNF↑	(Ref. 65)
	DM	C57BL/6 Mice[STZ]	Cognitive Dysfunction↓, GFAP↑, SYP↑	(Ref. 68)

Abbreviations: DM: diabetes mellitus; AD: Alzheimer’s Disease; HFD: high-fat diet; STZ: streptozotocin; FBG: fasting blood glucose; HbA1c: haemoglobin A1c; Glu: glucose; GSIS: glucose-stimulated insulin secretion; IRS: insulin receptor substrate; PI3K: phosphoinositide 3-kinase; AKT: protein kinase B; SOD2: superoxide dismutase 2; TRX2: thioredoxin 2; PRDX3: peroxiredoxin-3; NF-κB: nuclear factor-κB; TNF-α: tumour necrosis factor-α; IL-1β: of interleukin-1 beta; IL-6: interleukin- 6; NEP: neprilysin; Aβ: amyloid β-protein; BACE1: beta-secretase 1; BDNF: brain-derived neurotrophic factor; GFAP: glial fibrillary acidic protein; SYP: synaptophysin.

### Energy metabolism

Disruption in energy metabolism can lead to brain region damage, contributing to cognitive impairment. Indeed, energy metabolism disturbances in ageing, AD and DACD are multifactorial. First, reduced brain glucose metabolism is a hallmark feature of cognitive impairment, where impaired glucose uptake in the brain can lead to brain atrophy and neuronal dysfunction. Irisin, by promoting the browning of white adipose tissue, improves glucose metabolism and effectively regulates systemic energy metabolism (Ref. 41). Second, mitochondrial dysfunction forms the basis of brain injury and cognitive impairment. Mitochondria are crucial organelles responsible for energy metabolism, with neurons having higher mitochondrial concentrations compared to other cells, which is essential for maintaining electrical and synaptic transmission (Ref. 42). Irisin can induce mitochondrial biogenesis and mitochondrial uncoupling to improve cognitive impairment.

A clinical cross-sectional study has shown that genetic variations in FNDC5 are associated with reduced brain glucose metabolism in patients with cognitive impairment, suggesting that FNDC5 may participate in brain metabolic regulation in regions susceptible to AD pathology (Ref. 43). in vivo studies indicate that irisin injection increases energy expenditure and improves glucose metabolism. Intraperitoneal injection of recombinant irisin dose-dependently reduces blood glucose in insulin-deficient diabetic mice, potentially through improved function of skeletal muscle and white adipose tissue, characterized by activation of energy and metabolism-related genes (Ref. 44). Additionally, short-term treatment with irisin in obese mice improves glucose homeostasis and results in modest weight loss (Ref. 2). The lack of FNDC5/irisin may induce dysregulation in mitochondrial dynamics and bioenergetics, whereas exogenous irisin injection can protect against brain injury by enhancing mitochondrial quality control mediated by SIRT3 (Ref. 45). Previous evidence strongly suggests that irisin enhances mitochondrial function by regulating the mitochondrial inner membrane transport protein uncoupling protein 2 (UCP2), demonstrating potential neuroprotective effects (Refs. 46, 47). Upregulation of UCP2 expression may contribute to maintaining BBB integrity and a normal brain environment. Furthermore, irisin, regulated by PGC1-α, is secreted from muscles into circulation, where moderate increases in circulating irisin induce browning of white adipose tissue and increase UCP1 expression (Ref. 2). Studies also suggest that irisin regulates glutathione peroxidase 4 via nuclear factor-erythroid 2-related factor 2, thereby inhibiting ferroptosis in the hippocampus and improving mitochondrial dysfunction (Ref. 48). Despite these findings, the potential mechanisms by which irisin regulates energy metabolism to improve cognitive impairment are intricate and influenced by numerous factors. Further in-depth research is needed to comprehensively elucidate how irisin modulates energy metabolism in cognitive impairment-related diseases.

### Insulin resistance

A large body of studies confirms that brain insulin resistance may be a potential mediator in the development and progression of cognitive dysfunction, particularly in AD and DACD. Another mechanism through which irisin improves cognitive impairment involves its role in insulin signalling, especially in the context of insulin resistance. There is a strong correlation between FNDC5/irisin and insulin resistance. In humans, irisin is positively correlated with circulating insulin levels and β-cell function, such as HOMA and HOMA2, in individuals with normal glucose tolerance (Refs. 46, 49). Elevated circulating irisin levels may indirectly improve insulin resistance by reducing fasting insulin levels (Ref. 50), suggesting a role for irisin in modulating β-cell function. Research also indicates that while irisin correlates positively with fasting insulin levels, it does not correlate with postprandial insulin levels (Ref. 51). Basic studies have found that irisin alleviates β-cell insulin resistance through activation of the PI3K/AKT/FOXO1 signalling pathway (Ref. 52). Furthermore, recombinant irisin stimulated insulin biosynthesis and glucose-stimulated insulin secretion (GSIS) in a PKA-dependent manner and prevented apoptosis in human and rat pancreatic β-cells as well as in human and murine pancreatic islets (Ref. 53). Peripheral insulin resistance may disrupt brain insulin activity, which acts on neurons and glial receptors in cognition-related areas such as the cerebral cortex, olfactory bulb, hippocampus and hypothalamus. Dysregulation of this modulatory function may lead to impairment in various aspects of brain physiology and cognitive function (Refs. 54, 55). These findings underscore the importance of irisin in regulating insulin resistance and its potential positive effects on neuronal function and viability.

### Oxidative stress and inflammation

In recent years, a large body of research data supports the role of oxidative stress and inflammation in causing cognitive dysfunction by affecting the structure and function of the hippocampus, particularly in ageing, AD and DACD. Studies confirm that irisin can modulate inflammation and oxidative stress through multiple pathways, thereby improving cognitive impairments. First, irisin exhibits potent antioxidant properties by acting as a scavenger of free radicals to neutralize excess free radicals, thus reducing oxidative stress-induced damage to neurons. Second, the anti-inflammatory properties of irisin are related to cytokine regulation, which activates various signalling pathways and enhances the anti-inflammatory phenotype of glial cells, thereby playing an anti-inflammatory role. Recent research has found that FNDC5/irisin protects neurons by inhibiting pathways involving Caspase3 and Bax, increasing mitochondrial antioxidants in neuronal cell lines (NSC-34), and reducing cell apoptosis (Ref. 56). Additionally, irisin has been shown to prevent memory and cognitive deficits by reducing inflammation-induced damage in the brains of mice induced by STZ through modulation of the JAK/STAT and STAT3 signalling pathways (Ref. 33), as well as by attenuating lipotoxicity-induced inflammatory responses via inhibition of the TLR4/nuclear factor-κB (NF-κB) signalling pathway (Ref. 52). As we all know, activated glial cells produce various proinflammatory mediators that induce changes in the hippocampus involved in spatial learning and memory. Irisin crosses the BBB from the periphery into the central nervous system, effectively reducing levels of inflammatory factors such as TNF-α, IL-6 and IL-1β, and inhibiting the infiltration of microglia and monocytes into central nervous system inflammatory lesions (Refs. 57–59), accelerating the transformation of microglia from the M1 pro-inflammatory phenotype to the M2 anti-inflammatory phenotype. Furthermore, irisin not only promotes the phenotype transition of microglia but also inhibits M1 macrophage polarization and the production of inflammatory cytokines (Ref. 60), inducing JAK2-STAT6-dependent transcription to activate PPAR-γ-related anti-inflammatory systems and nuclear factor erythroid 2-related factor 2 (Nrf2)-related antioxidant genes to promote M2 macrophage differentiation (Ref. 61). Irisin also activates neuroprotective markers such as BDNF and CREB, as well as antioxidant markers like Nrf2/HO-1, while reducing the expression of inflammatory biomarkers (e.g., iNOS and COX-2) and beta-secretase 1 (BACE1) in the hippocampus and cerebral cortex of mice with cognitive impairments (Ref. 62). Interestingly, some studies have found that the expression of FNDC5 may be downregulated under inflammatory conditions, which may be related to the body’s attempt to maintain energy homeostasis by slowing down the browning of adipocytes.

Overall, irisin improves cognitive impairments by reducing pro-inflammatory cytokines, increasing anti-inflammatory cytokines and promoting M2 microglial polarization to prevent immune cell infiltration into brain tissues.

### Aβ deposition

In the brain, the intracellular aggregation of toxic proteins such as Aβ in plaque forms and hyperphosphorylated tau protein can lead to memory loss and other cognitive impairments. Clinical studies have found a significant positive correlation between cerebrospinal fluid levels of irisin and Aβ42 in patients with AD and a negative correlation with total tau protein levels (Refs. 38, 63). The mechanism by which irisin reduces Aβ deposition may involve the secretion of Aβ-degrading enzymes and cleavage sites of amyloid precursor protein (APP). Research using AD three-dimensional (3D) cell culture models suggests that irisin induces ERK-STAT3 signalling to regulate astrocytic release of neprilysin (NEP), which degrades Aβ (Ref. 64). Another study indicates that irisin strongly binds to specific structural domains between β-secretase and α-secretase cleavage sites of APP, inhibiting β-secretase expression or activity and promoting α-secretase-mediated proteolytic cleavage, thereby reducing Aβ production (Ref. 65). However, whether irisin’s inhibitory effect on Aβ production is mediated by peripherally produced irisin from external tissues or neuron-derived FNDC5/irisin remains unclear. Furthermore, some studies have found no significant correlation between irisin levels and Aβ40 or Aβ42 levels (Refs. 20, 66). Heterogeneous results are possibly due to the study cohort primarily comprising Asian individuals and the sample size is small. In view of the racial/ethnic heterogeneity recorded in dementia epidemiology, more surveys of different populations are needed to verify the universality of these findings in the future. In addition, previous studies have reported that the Aβ1–40 and Aβ1–42 levels in plasma compartments are not significantly correlated with those in CSF (Ref. 67); therefore, the correlation between irisin and Aβ40 or Aβ42 is also influenced by whether Aβ comes from CSF or plasma concentrations. Finally, the timing of irisin measurement, post-exercise irisin degradation during storage and variability in assay kit accuracy may also be the reasons for the heterogeneity (Ref. 19). In general, further research is needed to fully understand the potential mechanisms by which irisin reduces Aβ deposition and improves cognitive function.

### Synaptogenesis and plasticity

Irisin in circulation is capable of crossing the BBB, initiating hippocampal neuroprotective programmes, upregulating the expression of neurotrophic factors within the brain, promoting neurogenesis and protecting neurons from damage. These effects may contribute to enhancing synaptic connections between neurons, thereby improving cognition. BDNF is a hallmark of synaptic plasticity in the central nervous system, expressed in multiple brain regions including the frontal cortex and hippocampus. It promotes brain development, including neuronal survival, differentiation, migration, synaptic formation and plasticity (Ref. 68). Research indicates that age-related cognitive impairment is associated with decreased expression of PGC-1α, FNDC5 and BDNF in the hippocampus (Ref. 21). Similarly, clinical studies have found a positive correlation between cerebrospinal fluid irisin levels and BDNF (Ref. 38). Irisin and its precursor FNDC5 are associated with BDNF and positively correlated with cognition, especially memory function associated with the hippocampus (Ref. 37); knockdown of FNDC5/irisin in mouse brain cells impairs hippocampal memory function (Ref. 68). However, overexpression of FNDC5/irisin in primary cortical neurons increases BDNF expression, enhancing neuronal survival and supporting new neuronal growth and differentiation, significantly improving synaptic plasticity and memory function in mice (Ref. 68). Therefore, it is hypothesized that the association between irisin, BDNF and cognition suggests that BDNF mediates the effects of irisin on cognition. Circulating irisin reaching the brain enhances BDNF expression, which is crucial for hippocampal synaptic function and memory, benefiting the central nervous system. Interestingly, studies have also found a positive correlation between hippocampal BDNF levels in rats and serum irisin but not with hippocampal FNDC5/irisin (Ref. 69). Hence, further research is needed to clarify whether irisin’s role in improving cognitive impairment operates primarily via its actions in serum or within the brain.

## Signalling pathways of irisin in improving cognitive impairment

**Figure 3.**
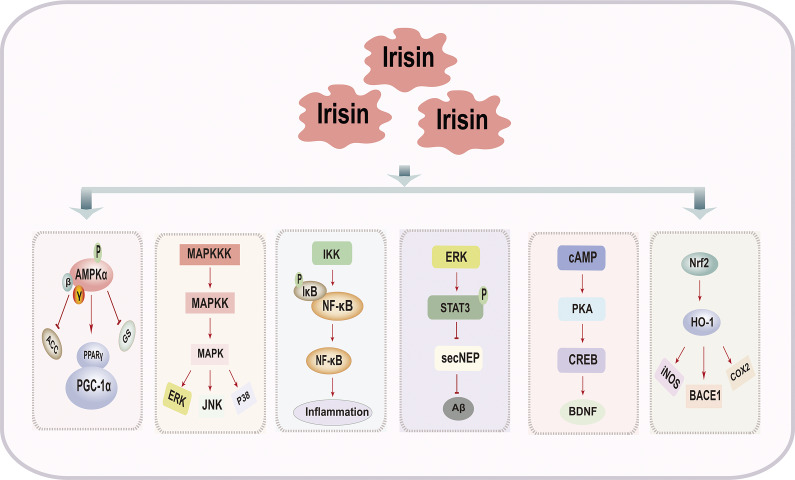
Signaling Pathways of Irisin in Improving Cognitive Impairment.

The signalling pathways through which irisin improves cognitive impairment are complex and highly regulated processes, involving multiple signalling pathways such as the adenosine monophosphate-activated protein kinase (AMPK) signalling pathway, mitogen-activated protein kinase (MAPK) signalling pathway, NF-κB signalling pathway, ERK-STAT3 signalling pathway, cAMP/PKA/CREB signalling pathway and Nrf2/HO-1 signalling pathway (Figure 3 and Table 2).

**Table 2.** Signalling pathways of irisin in improving cognitive impairment

Pathway	Activation(+) or inhibition(−)	Disease	Model	Phenomenon	References
AMPK	+	ICH	C57BL/6 mice	IL–1β↓, TNF-α↓, MPO↓, Bax	(Ref. 74)
MAPK	−	DM	C2C12 cells	G6Pase↓, PEPCK↓, GLUT–4↓, glucose utilization↓	(Ref. 81)
NF-κB	−	DM	C57BL/6 mice[HFD] + HUVECs[HG/HF]	superoxide↓, iNOS↓	(Ref. 86)
ERK-STAT3	−	AD	3D cell culture model	NEP↑, Aβ↓ IL–2↓, IL–6↓	(Ref. 64)
cAMP/PKA/CREB	+	AD	APP/PS1 ΔE9 mice	LTP↑, BDNF↑	(Ref. 95)
Nrf2/HO–1	+	Aging	C57BL/6 J mice[LPS] + BV–2 microglial	Nrf2↑, HO–1↑ iNOS↓, COX–2↓, BACE1↓	(Ref. 62)

Abbreviations: AMPK: adenosine monophosphate-activated protein kinase; AD: Alzheimer’s Disease; MAPK: mitogen-activated protein kinase; DM: diabetes mellitus; G6Pase: glucose-6-phosphatase; HUVECs: human umbilical vein endothelial cells; PEPCK: phosphoenolpyruvate carboxykinase; GLUT-4: glucose transporter 4; NEP: neprilysin; Aβ: amyloid β-protein; IL-1β: of interleukin-1 beta; IL-6: interleukin- 6; IL-2: interleukin- 2; TNF-α: tumour necrosis factor-α; MPO: myeloperoxidase; Bax: bcl-2-associated X protein; iNOS: inducible nitric oxide synthase; LTP: long-term potentiation; BDNF: brain-derived neurotrophic factor; Nrf2: nuclear factor erythroid 2-related factor 2; COX-2: cyclooxygenase-2; BACE1: beta-secretase 1.

### AMPK signalling pathway

AMPK is an intracellular signalling pathway that primarily functions to maintain cellular energy balance and regulate cellular metabolism (Ref. 70). Extensive research indicates that activation of the AMPK signalling pathway can improve AD-like pathology as well as spatial learning and memory deficits (Refs. 71–73). Upregulation and phosphorylation of AMPK activate PGC1α, leading to increased FNDC5 expression and irisin release (Ref. 74). Concurrently, FNDC5/irisin, through AMPK-mediated polarization of microglia/macrophages, suppresses the expression of pro-inflammatory cytokines IL-1β and TNF-α, thereby ameliorating neuroinflammation (Ref. 74). Additionally, overexpression of irisin enhances AMPKα phosphorylation, activating the AMPK signalling pathway, which improves β-cell dysfunction, reduces cellular apoptosis and alleviates insulin resistance (Refs. 30, 60, 75). It is important to note that when evaluating AMPK activation, consideration of AMPK subtypes is crucial, as assessing total cellular AMPK activity may obscure subtle yet significant differences.

### MAPK signalling pathway

The MAPK signalling pathway influences various fundamental cellular processes such as gene expression, mitosis, differentiation, apoptosis and stress responses (Refs. 76, 77). This pathway plays a central role not only in neuronal plasticity and the regulation of synaptic efficacy in long-term changes such as long-term potentiation (LTP) and long-term depression (LTD) but also in insulin resistance and regulation of neuroinflammatory responses (Refs. 78–80). Irisin exerts therapeutic potential against insulin resistance by inhibiting the p38 MAPK signalling pathway, enhancing glucose uptake and improving mitochondrial function and respiration (Refs. 81, 82). Additionally, studies have indicated that irisin can protect brain neurons by inhibiting the activation of the MAPK signalling pathway, thereby suppressing the expression of pro-inflammatory cytokines IL-6, IL-1β and TNF-α (Ref. 83). The MAPK signalling pathway serves as a potential downstream pathway through which irisin may improve cognitive impairment, playing a significant role in anti-inflammatory and neuroprotective effects, thus warranting further investigation in the future.

### NF-κB signalling pathway

The NF-κB is an important transcription factor involved in regulating various biological processes including immunity, inflammatory responses and apoptosis (Ref. 84). This family consists of NF-κB1 (p105/p50), NF-κB2 (p100/p52), RelA (p65), RelB and c-Rel (Ref. 85). As previously discussed, neuroinflammation, oxidative stress and apoptosis are key processes contributing to cognitive impairment. Therefore, the inflammation mediated by the NF-κB signalling pathway has been extensively studied in cognitive-related disorders. Research indicates that irisin attenuates neuroinflammatory responses in BV-2 microglial cells induced by LPS by inhibiting the NF-κB signalling pathway (Ref. 62). Furthermore, irisin regulates the expression of Matrix Metalloproteinase-9 (MMP-9) by inhibiting NF-κB phosphorylation, thereby reducing BBB permeability (Ref. 86). Additionally, irisin partially alleviates endothelial dysfunction in type 2 diabetes by inhibiting the NF-κB signalling pathway, potentially improving cognitive impairment associated with diabetes (Ref. 87). These findings suggest that irisin may improve cognitive impairment through the NF-κB inflammatory signalling pathway, although the exact mechanisms remain to be elucidated.

### ERK-STAT3 signalling pathway

Extracellular signal-regulated kinase (ERK) is a critical signalling pathway within cells, playing a key role in regulating various biological processes such as cell growth, differentiation, survival and metabolism. (Ref. 88). STAT3 is associated with hippocampal neurogenesis and is a key factor involved in many cytokine cascade reactions, including IL-6, IL-10 and TNF-α. (Ref. 89). ERK can regulate the activity of STAT3 either directly or indirectly. Research indicates that irisin improves the dysregulation of hepatic glucose/lipid metabolism under insulin-resistant conditions by stimulating ERK 1/2 phosphorylation (Ref. 90). It also prevents amyloid-beta oligomer-induced oxidative stress in primary hippocampal neurons (Ref. 90). In addition, irisin increases proliferation of mouse neuronal cells H19–7 HN by modulating neurogenesis-related STAT3 signalling, influencing hippocampal neurogenesis. (Ref. 91). Recent research has clarified that in AD models, irisin exerts its cognitive improvement effects by inhibiting the ERK-STAT3 signalling pathway (Ref. 64). This inhibition leads to increased secretion of soluble NEP from astrocytes, thereby reducing Aβ levels. Additionally, irisin decreases inflammatory factors, contributing to its role in improving cognitive impairment (Ref. 64). Therefore, the ERK-STAT3 pathway mediated by irisin is crucial in the pathophysiology of cognitive impairment-related disorders. Activating this pathway can slow disease progression and improve cognitive decline.

### cAMP/PKA/CREB signalling pathway

As is well known, the cAMP/PKA/CREB signalling pathway is associated with neural plasticity and its protective effects, playing a significant role in processes such as neural regeneration, learning, memory and emotional states. It has emerged as a crucial target for treating various central nervous system disorders, including neurodegenerative diseases (Refs. 92–94). Research has indicated that ex vivo adult cortical slices expressing FNDC5/irisin, as well as recombinant irisin, can activate the cAMP/PKA/CREB signalling pathway in human cortical slices (Ref. 95). Irisin promotes the generation of brain-derived neurotrophic factor via the cAMP/PKA/CREB pathway, interrupts the binding of AβO to neurons, thus preserving synaptic plasticity in the brains affected by AD, and promoting neurogenesis and dendritogenesis (Ref. 95). Further in-depth and systematic research is still needed to elucidate how irisin precisely regulates the cAMP/PKA/CREB signalling pathway and its specific mechanisms in improving cognitive impairments.

### Nrf2/HO-1 signalling pathway

Nrf2 is a transcription factor involved in regulating oxidative stress responses (Ref. 96). HO-1, a downstream target gene of Nrf2, helps neutralize oxidative stress within cells (Ref. 97). The Nrf2/HO-1 signalling pathway regulates antioxidant and anti-inflammatory responses by directly enhancing the clearance of excess Reactive Oxygen Species (ROS) and indirectly inhibiting cytokine production (Ref. 97). Research suggests that irisin acts as a regulator of the Nrf2/HO-1 pathway (Ref. 98). FNDC5/irisin can activate the antioxidant markers Nrf2/HO-1, which not only suppresses neuroinflammation mediated by microglial cells such as iNOS and COX-2 but also reduces the expression of BACE1 in the hippocampus and cerebral cortex of mice with cognitive impairments (Ref. 62). These findings provide a theoretical basis for irisin as a potential strategy to improve cognitive impairment-related diseases by modulating the Nrf2/HO-1 signalling pathway. However, further research and validation are needed to explore the specific clinical applications and therapeutic effects.

## Challenges in clinical translation of Irisin for neurodegenerative diseases

### Therapeutic challenges

Indeed, while irisin has shown promising effects in the context of neurodegenerative diseases in research, there are several challenges to its clinical application that need to be addressed. A primary obstacle lies in therapeutic delivery methods, as current approaches predominantly rely on subcutaneous or intravenous injections of recombinant irisin (Ref. 99), requiring frequent administration to maintain effective plasma concentrations. This limitation underscores the need for developing sustained-release systems or nanotechnology-based carriers to enhance bioavailability and delivery precision. The second is the stability of irisin. As a polypeptide hormone, the stability of irisin in vivo is affected by many factors. Studies have shown that its half-life in a physiological environment is short, which limits its durability as a therapeutic drug (Ref. 100). Future research needs to explore how to prolong the half-life of irisin by chemical modification or developing protective carriers, so as to improve its stability in vivo. Furthermore, safety considerations add another layer of complexity – while animal models demonstrate good tolerability, potential human risks such as immune activation at high concentrations, unintended metabolic interference and long-term tolerance development require thorough evaluation through clinical trials. Finally, to better evaluate the therapeutic potential of irisin, future research needs to focus on the following aspects: Clarify the receptor of irisin and its mechanism of action. At present, the receptor of irisin has not been completely clarified, which limits the further development of its clinical application. Carry out long-term clinical trials: there is a lack of long-term human studies on recombinant irisin therapy, which needs to be supplemented in future studies. Explore alternative delivery methods: besides injection, developing oral or other non-invasive delivery methods may be the future research direction.

### The role of gender differences in irisin’s effects on cognitive impairment

Interestingly, recent studies have found lower cerebrospinal fluid irisin levels in patients with AD, with a negative correlation between irisin levels and total tau observed only in female patients, as well as a negative correlation with the Clinical Dementia Rating-Sum of Boxes (CDR-SOB) (Ref. 63). In addition, previous research reported that irisin levels were higher in young women than in men after adjustment for lean body mass (Ref. 101). Similarly, a cross-sectional study reported that the level of circulating irisin in girls was higher than that in boys (Ref. 102). The gender-specific influence of irisin may be explained by hormonal differences. Oestradiol, which is an anabolic hormone, has been positively correlated with irisin among female adults and may influence irisin circulation through anabolic pathways to increase muscle mass, leading to irisin upregulation (Refs. 101, 102). Apart from this, the difference in the distribution of brown and white adipose tissue between the sexes may also be the cause of gender differences in irisin’s effects on cognitive impairment because the distribution of these tissues is known to be sexually dimorphic (Refs. 103, 104). Therefore, we suggest that gender should be considered as an important variable in future research, so as to understand the role of irisin in cognitive impairment and neurodegenerative diseases more comprehensively.

### Standardized measurement

As a new therapeutic target of cognitive impairment, irisin needs reliable detection methods. At present, the measurement methods of irisin mainly include enzyme-linked immunosorbent assay (ELISA), Western blot and mass spectrometry (MS). However, there are significant differences in sensitivity and specificity between these methods, which lead to inconsistent measurement results. For example, early studies based on ELISA reported extremely high levels of irisin (from a few micrograms to dozens of micrograms/millilitre) (Refs. 105, 106), while more accurate mass spectrometry measurements showed that the reference level of irisin in human serum was only 3.6–4.3 ng/mL (Ref. 6). To standardize the detection method of irisin, the key lies in developing a highly sensitive and specific ELISA kit, establishing a consistent mass spectrometry analysis process, using standardized reference materials and cross-laboratory verification. At the same time, future research should promote the application of automation and Qualcomm detection technology to improve the reliability and repeatability of data. The improvement of these methods will help to ensure the accuracy of irisin-level measurement and promote its wide application in physiological and pathological research.

## Conclusion and future prospects

Irisin is regarded as a novel neuroprotective factor with promising potential to improve cognitive function, demonstrating significant application prospects. Its role in combating cognitive deficits is associated with the regulation of energy metabolism, insulin resistance, Aβ deposition, inflammation, oxidative stress and synaptic plasticity. Given irisin’s broad functionality in treating cognitive impairment-related diseases, pharmacologically increasing brain irisin levels may represent a new therapeutic strategy to prevent age-related cognitive decline, AD and DACD. However, it should be emphasised that irisin as a potential treatment strategy for cognitive disorders, is still in its early stages. First, the lack of reliable measurement techniques for endogenous irisin raises uncertainties about its prognostic potential in improving cognitive impairment, and it is necessary to use standardized methods to measure the irisin level. Second, the identification of irisin cell receptors is awaited, limiting our current understanding of downstream signalling mechanisms. Finally, while numerous studies have shown irisin’s potential in improving brain cognitive impairments in animal models, its exact protective effects require large-scale clinical trial data to validate its safety and efficacy and to determine its dosage range and optimal treatment window in clinical applications. Therefore, future research needs to further elucidate the close relationship between irisin and the brain and explore in depth the potential value of irisin in treating age-related cognitive impairments, AD and diabetes-related cognitive disorders.

## Abbreviations

Aβ: amyloid β-protein;
AD: Alzheimer’s disease;
AMPK: adenosine monophosphate-activated protein kinase;
APP: amyloid precursor protein;
BBB: blood–brain barrier;
BDNF: brain derived neurotrophic factor;
DM: diabetes mellitus;
FNDC5: Fibronectin type III domain-containing 5;
3D: three-dimensional;
DACD: diabetes-associated cognitive dysfunction;
GSIS: glucose-stimulated insulin secretion;
Hsp90: heat shock protein-90;
IL-1β: Interleukin-1 beta;
IL-6: Interleukin- 6;
IST: Isaac’s Set Test;
LTD: long-term depression;
LTP: long-term potentiation;
MAPK: signalling pathway, mitogen-activated protein kinase;
MMSE: Mini-Mental State Examination;
NEP: neprilysin;
Nrf2: nuclear factor erythroid 2-related factor 2;
NSC-34: neuronal cell lines;
OXPHOS: oxidative phosphorylation;
RALVT: Rey Auditory Verbal Learning Test;
T2DM: type 2 diabetes mellitus;
TFAM: mitochondrial transcription factor A;
TNF-α: Tumour Necrosis Factor-α;
UCP2: uncoupling protein 2.
